# Australian First-Year Nursing Student Knowledge and Attitudes on Pressure Injury Prevention: A Three-Year Educational Intervention Survey Study

**DOI:** 10.3390/nursrep12030042

**Published:** 2022-06-22

**Authors:** Carey Mather, Angela Jacques, Sarah J. Prior

**Affiliations:** 1 School of Nursing, College of Health and Medicine, University of Tasmania, Launceston, TAS 7250, Australia; 2 Institute for Health Research, University of Notre Dame Australia, Fremantle, WA 6959, Australia; angela.jacques@nd.edu.au; 3 Tasmanian School of Medicine, College of Health and Medicine, University of Tasmania, Burnie, TAS 7320, Australia; sarah.prior@utas.edu.au

**Keywords:** attitudes, higher education, first-year student, knowledge, nursing, pressure injury, prevention, wound

## Abstract

Pressure injury prevention is a significant issue as pressure injuries are difficult to heal, painful, and create clinical complications for patients. The aim of this study was to investigate knowledge and attitudes of first-year nursing students to pressure injury prevention, and to explore whether additional educational interventions augmented learning. A previously validated online survey was administered to three cohorts of first-year nursing students in 2016, 2017 (after additional online education), and 2018 (after further simulation education), and a subsequent comparative analysis was undertaken. Overall, the knowledge of students about pressure injury was low with measures to prevent pressure injury or shear achieving the lowest score (<50%). Students aged over 25 years (*p* < 0.001) and men (*p* = 0.14) gained higher attitude scores. There were significant differences for mean knowledge scores between the 2016 and 2018 cohorts (*p* = 0.04), including age group (*p* = 0.013) and number of clinical training units undertaken (*p* = 0.23). The 2016 cohort scored consistently lower in the attitude survey than both other cohorts (*p* < 0.001). Online resources and simulation experiences marginally improved knowledge and improved attitudes towards prevention of pressure injury. Nursing curricula should include targeted education to ensure student nurses are adequately prepared to prevent pressure injury through understanding of aetiology and risk assessment.

## 1. Introduction

Pressure injuries are localised areas of tissue breakdown in skin and/or underlying tissues as a result of a sustained mechanical loading [1]. Pressure injuries are a significant issue in many health and social care environments with clinical complications for recipients of care (known as patients in this survey context), quality issues, and financial implications for organisations [2,3,4]. Treatment for pressure injuries costs two and a half times more than prevention [5]. Additionally, high costs of care associated with pressure injuries can also interfere with functional recovery, be complicated by infection and pain, and can also increase hospital length of stay [6,7]. Identification of pressure injuries is a nurse-initiated patient safety action [8] heavily reliant on visual assessment and clinical judgement.

Pressure injury incidence is widely accepted as an indicator for quality of care [9]. Poor knowledge and negative attitudes towards pressure injury prevention undesirably affect preventive care strategies [3,10]. Although international [1,9] and national [11] guidelines have been developed to reinforce the importance of prevention and care of pressure injuries, nurses continue to have a range of knowledge about pressure injury and its prevention. A barrier for implementation of evidence-based practice of pressure injury is a lack of appropriate education and theoretical knowledge [12] in the undergraduate study pathways, which often focus on acute surgical trauma or chronic disease wound management rather than preventing hospital-acquired wounds. Nursing students often do not have sufficient knowledge about contemporary guidelines, preventing utilisation once they begin practicing in healthcare environments [3,7,12]. It is important for nursing students to have sufficient knowledge, skills, and positive attitudes on completion of educational preparation to prevent pressure injuries, and if necessary, to recognise, assess, and treat these wounds properly [8,13]. It is important to identify widely spread misconceptions and evaluate educational needs about pressure injury prevention to develop strategies for improving the quality of pressure injury education and prevention. The aim of this study was to investigate whether supplementing curricula with additional educational interventions changed pressure injury prevention knowledge and attitudes of nursing students enrolled at one university and educated in two states in Australia over a three-year period. Baseline knowledge and attitudes were investigated with further online and simulation educational interventions introduced in two subsequent years to augment educational preparation about pressure injury.

## 2. Materials and Methods

### 2.1. Study Design

A cross-sectional study design was utilised to collect population-based data through supervised, paper-based self-report questionnaires. Data were collected from participants at the same point in educational preparation each year from three individual cohorts of first-year nursing students over three consecutive years between 2016 and 2018.

### 2.2. Population

A convenience sample of nursing students was surveyed using two validated questionnaires [14,15] to assess knowledge and attitudes on pressure injury prevention. The inclusion criteria included all first-year nursing students at one university in two states in Australia between 2016 and 2018. Students were invited to participate in the study and complete the survey at the conclusion of a routine simulation session on wound management. Students self-selected and students from other years of study were excluded from the research as they were further through their course and had undertaken more clinical training units than first-year students. An independent third party explained the study to students and provided information sheets. Students who wished to participate were given a consent form and questionnaire and time to complete the survey. Participants were supervised whilst completing the questionnaire to ensure they did not refer to textbooks, notes, the internet, or confer with friends. Completed consent forms and questionnaires were collected in separate sealed boxes to ensure participant privacy and confidentiality.

The three cohorts of students in the study included:

- Students in the 2016 cohort that were considered a baseline cohort as they were prepared with guidance from the Australian Nursing and Midwifery Accreditation Council Standards [16]. Students received 10 h of online learning content which included theory and activities related to wound development, management, evaluation, and nursing interventions including wound dressings. To scaffold content and consolidate student learning, a two-hour simulation workshop was undertaken by students. Activities included site and wound assessment, understanding different types of dressings for wounds, and introduced to wound dressing techniques. The survey was distributed at the end of this session. Participants were part of a national study [8]. Ethical approval was gained from the Human Research Ethics Committee (Tasmania) Network prior to commencement of the national study and amended to accommodate the educational interventions in 2017 and 2018 (H0015798).
- In 2017, an additional online learning and teaching intervention was included into the first-year nursing content. The ‘Stop the Pressure’ online learning tool [17] was chosen by the lecturer in charge of the unit of study. When introduced in eastern England, a 50% reduction in the incidence of new pressure injuries was reported. The survey was repeated and a comparison with previous scores evaluated the effectiveness of this intervention and provided direction for further educational development of pressure injury prevention learning content within the simulation environment [18].
- In 2018, additional image-based educational learning was provided within a simulation environment, which aimed to consolidate information available in the online learning tool [17].

### 2.3. Survey Instruments

#### 2.3.1. Knowledge Assessment Instrument

The Knowledge Assessment Instrument [15] is a validated questionnaire to assess knowledge of pressure injury prevention. The survey consists of 26 multiple choice items and three alternative responses reflecting 6 themes expressing the most relevant aspects of pressure injury prevention: (1) aetiology and development; (2) classification and observation; (3) nutrition; (4) risk assessment; (5) reduction in the magnitude of pressure and shearing; and (6) reduction in the duration of pressure and shearing. Total possible knowledge score of 25 points can be achieved.

#### 2.3.2. Attitude toward Pressure Ulcer (APuP) Tool

The APuP [14] is a 13-item questionnaire that measures subjective attitudes toward pressure injury prevention. The questionnaire comprises five subscales: (1) personal competency to prevent pressure injuries, (2) priority of pressure injury prevention, (3) impact of pressure injuries, (4) responsibility in pressure injury prevention, and (5) confidence in the effectiveness of prevention. Responses were scored on a 4-point Likert-type scale (1 = strongly disagree, 2 = disagree, 3 = agree, and 4 = strongly agree). Attitude scores—a higher score indicates higher empathy towards pressure injury prevention. Demographic and general information was also collected from participants: including age, sex, and current year of nursing program. Students were asked to provide information about previous clinical experiences.

### 2.4. Statistical Methods

Sample descriptive data were summarised using frequency distributions for categorical variables and means and standard deviations for continuous data. Item answers and correct answers were summarised using frequency distributions. Sum questionnaire scores for knowledge and attitude questionnaires, for participants that answered a minimum of 24 and 12 questions, respectively, were summarised using means and standard deviations, and overall comparisons were made between demographic variable categories using one-way ANOVA. Linear regression models were used to assess the impact of demographic and educational factors on knowledge and attitude scores, adjusting for cohort year, age, and sex. Model fit was assessed by checking residuals graphically. Data were analysed using IBM SPSS version 24.0 [19]. *p*-values < 0.05 were considered statistically significant.

## 3. Results

### 3.1. Demographic Data

One thousand one hundred and two first-year nursing students completed the questionnaire between 2016 and 2018. Most (84%) of these students were women and 38% were aged over 25 years. Only 9% of students had completed more than one clinical training unit at the time they completed the survey and 79% had completed no clinical training units. The demographic data are shown in Table 1. There were significant differences in the age groups of students in each cohort, with 2017 having a higher number of students >25 years and 2018 having a higher number of students under the age of 20 years. There was also a significant difference between the number of clinical training units undertaken and clinical experiences, with students in 2016 having less clinical time than students in 2017 and 2018 (*p* =< 0.001).

### 3.2. Knowledge

Overall, knowledge of first-year nursing students for pressure injury prevention between 2016 and 2018 was low, with a mean correct response rate of 51.7% (12.87/25). The mean knowledge scores across each cohort are shown in Table 2. Significant differences were noted for overall mean knowledge scores between the 2016 and 2018 cohorts (*p* = 0.04), age group and mean knowledge scores (*p* = 0.013), and number of clinical training units undertaken and mean knowledge scores (*p* = 0.023). The responses for individual themes are discussed below.

#### 3.2.1. Theme 1 (Pressure Injury Aetiology and Development)

Overall, the majority of participants responded incorrectly for items in Theme 1 (Table 3). On average, 19% of participants correctly identified that a lack of oxygen causes pressure injuries (Item 1), 40% of participants correctly identified that extremely thin patients are more at risk of developing pressure injuries than obese patients (Item 2), 39% of participants correctly identified the outcomes of patients sliding down the bed (Item 3), and 48% of participants correctly identified what shear is (Item 4). Most participants, on average, identified correctly that pressure injury risk increases with recent weight loss (71%) (Item 5) and that there is no relationship between pressure injury and hypertension (68%) (Item 6). The participants in the 2016 cohort consistently responded with less correct answers than the 2017 and 2018 cohorts.

The number of correct responses was significantly lower in the 2016 cohort for Item 2 when compared to the 2018 cohort (*p* < 0.001), and when compared to the 2017 and 2018 cohorts for Item 5 (*p* = 0.030).

#### 3.2.2. Theme 2 (Pressure Injury Classification and Observation)

Overall, many participants provided correct responses for three out of five items in Theme 2 (Table 4). On average, 53% of participants correctly identified the grade of pressure injury when necrosis occurs (Item 2), 82% of participants correctly identified that friction or shear may occur when moving a patient in bed (Item 3), and 59% of participants correctly identified that pressure injuries are most likely to occur on the pelvic area, elbow, and heel (Item 4). On average, only 27% of participants correctly identified what a grade 3 pressure injury is (Item 1), and only 21% of participants correctly identified the needs of patients with heel pressure injuries (Item 5).

The 2016 cohort provided less correct responses than the 2017 and 2018 cohorts for three out of five items and less correct responses than the 2018 cohort for four out of five items. There was a significantly higher correct response rate for the 2017 cohort for Item 2 when compared with the 2016 and 2018 cohorts (*p* = 0.012). However, there was a significantly lower correct response rate for the 2018 cohort for Item 4 when compared to the 2016 and 2017 cohorts (*p* = 0.027).

#### 3.2.3. Theme 3 (Pressure Injury Risk Assessment)

Overall, the majority of participants correctly responded to both questions in Theme 3 (Table 5). On average, 69% of participants correctly identified that a risk assessment scale may not accurately predict the risk of developing pressure injuries (Item 1), and 58% of participants correctly identified that patients with a history of pressure injuries are at higher risk of developing new pressure injuries (Item 2).

Although there were no significant differences noted in the number of correct responses across the three cohorts, participants in the 2016 cohort consistently provided less correct responses than participants in the 2018 cohort.

#### 3.2.4. Theme 4 (Pressure Injury and Nutrition)

Overall, the majority of participants correctly responded to the item in Theme 4 (Table 6). On average, 87% of participants correctly identified that optimising nutrition may reduce the risk of pressure injuries (Item 1). There was no significant difference between the number of correct responses between the cohorts.

#### 3.2.5. Theme 5 (Preventative Measures to Reduce the Amount of Pressure or Shear)

Overall, many participants correctly responded to two out of seven items in Theme 5 (Table 7). On average, 73% of participants correctly responded that patients who can change position while sitting should be taught to shift their weight at minimum every 60 min while sitting (Item 3), and 52% of participants correctly identified the main disadvantage of water mattresses (Item 6). On average, only 33% of participants correctly identified the sitting position with the lowest amount of pressure between the body and the seat (Item 1), 32% of participants correctly identified the repositioning scheme that reduces pressure injury risk the most (Item 2), 28% of participants correctly identified that a thick air cushion reduces the magnitude of pressure for a patient sliding down a chair (Item 4), 40% identified the correct use of a visco-elastic foam mattress (Item 5), and 36% of participants identified correctly that elevation of the heels is necessary when a patient is lying on a pressure-reducing foam mattress (Item 7).

The 2016 cohort provided more correct responses to Item 1 when compared to both the 2017 and the 2018 cohorts. However, correct responses were significantly lower for the 2018 cohort for Item 1 (*p* = < 0.001) when compared to the responses from both the 2016 and 2017 cohorts.

#### 3.2.6. Theme 6 (Preventative Measures to Reduce the Duration of Pressure or Shear)

Overall, the majority of participants responded correctly to three out of five items in Theme 6 (Table 8). On average, 74% of participants correctly identified that fewer patients would develop a pressure injury if patients were mobilised (Item 2), 53% of participants correctly identified that patients at risk lying on a non-pressure-reducing foam mattress should be repositioned every 2 h (Item 3), and 53% of participants correctly identified that patients should have a cushion under the lower legs elevating the heels when on an alternating pressure air mattress (Item 4). On average, only 35% of participants correctly identified why repositioning is an active preventative measure (Item 1), and 50% of participants correctly identified the most appropriate pressure injury prevention for patients who are bedridden and cannot be repositioned (Item 5). The 2016 cohort had more correct responses for Items 3, 4, 6, and 7 when compared to the 2017 and 2018 cohorts.

There was a significantly lower number of correct responses from participants in the 2018 cohort for Item 3 (*p* < 0.001) when compared with 2016 and 2017 cohorts. There were also significantly lower numbers of correct responses for the 2018 cohort for Item 5 (*p* = 0.029) when compared with the 2016 responses, but no significant difference when compared to the 2017 cohort.

### 3.3. Attitudes

Overall, there were significant differences between the mean attitude scores for the 2016 and the 2017 and 2018 cohorts (*p* < 0.001), suggesting that the 2016 cohort consistently scored lower on attitude compared to the 2017 and 2018 cohorts. There was a significant difference between mean attitudes scores by sex (*p* < 0.001), suggesting that men scored consistently higher across each cohort. There was a significant difference between age groups (*p* < 0.001), with those older than 25 years consistently scoring higher. There was also a significant difference between attitude scores and the number of clinical training units that students had completed (*p* = 0.014), with students who had completed one clinical training unit scoring higher than students who had completed zero clinical training units or more than one clinical training unit. The mean attitude scores across each cohort are shown in Table 9. 

The responses to statements about attitudes for first-year nursing students for pressure injury prevention differed inconsistently between year cohorts across the five themes (Table 10). Each theme is also discussed individually below.

#### 3.3.1. Theme 1: Competency

Self-reported competency was generally high across all three cohorts. The majority of participants, on average, reported that they were confident in their ability to prevent pressure injuries (Item 1) (60%), that they felt well-trained to prevent pressure injuries (Item 2) (51%), and that they do not believe pressure injury prevention to be too difficult (Item 3) (60%).

There was a significant difference in reported competency between participants in the 2016 cohort and participants in the 2017 and 2018 cohorts for Item 1 (*p* = 0.015), with participants in the 2016 cohort feeling less confident in their ability to prevent pressure injuries. There was also a significant difference in reported competency between participants in the 2016 cohort and participants in the 2018 cohorts for Item 2 (*p* < 0.001), with participants in the 2016 cohort indicating that they felt they were less educationally prepared to prevent pressure injuries compared to those in the 2018 cohort.

#### 3.3.2. Theme 2: Priority

Overall, many participants across all cohorts indicated that pressure injury prevention is important and should be a priority. On average, only 15% of participants indicated that they think too much attention goes into pressure injury prevention (Item 1), and 6% felt that pressure injury prevention is not that important (Item 2), with 94% of participants believing that pressure injury prevention should be a priority (Item 3).

A significant difference in reported priority was noted between the 2016 and 2018 cohorts for Item 2 (*p* = 0.037), where more participants in 2016 felt that pressure injury prevention is important compared with participants in 2018.

#### 3.3.3. Theme 3: Impact

The majority of participants overall agreed that the impact of pressure injuries is felt by patients and society. On average, 94% of participants agreed that pressure injuries cause discomfort for patients, 52% agreed that financial impact of pressure injuries on patients should not be exaggerated, and 81% agreed there is a high financial impact of pressure injuries on society.

A significant difference in responses was noted between the 2016 cohort and the 2018 cohort for Item 1 (*p* = 0.010), with the 2018 cohort responding less often that pressure injuries never cause discomfort to patients than the 2016 cohort. A significant difference in responses was reported between the 2016 and the 2017 and 2018 cohorts for Item 3 (*p* = 0.004), with the 2016 cohort indicating less agreeance that the societal impact of pressure injuries is high.

#### 3.3.4. Theme 4: Responsibility

Overall, the majority of participant responses aligned with taking responsibility for pressure injury prevention. On average, 90% of participants indicated that they would be responsible if their patient developed a pressure injury (Item 1), and 96% indicated they believed nurses have an important role in pressure injury prevention (Item 2). There was no significant difference in responses for any items between the cohorts.

#### 3.3.5. Theme 5: Confidence

Overall, most participants agreed that pressure injuries are preventable in high-risk patients (91%) (Item 1) and that pressure injuries are preventable (90%) (Item 2). There was no significant difference noted in responses for any items between the cohorts.

## 4. Discussion

The aim of this study was to assess whether supplementing curricula with additional educational interventions changed knowledge and attitudes of first-year nursing students about pressure injury prevention. Poor knowledge and attitudes toward pressure injury prevention is a common issue in nursing [12,20,21,22], and this deficit has been shown to directly affect patient outcomes [2,4] and staff experience [3,5]. Barakat-Johnson [23] suggested that nurses are aware of the importance of pressure injury prevention and management but find it difficult to translate this positive attitude into quality, evidence-based care due to competing priorities and challenges at an organisational or individual care level [7]. With sub-optimal knowledge and little time, sound education at the foundational level is important to ensure pressure injury prevention guidelines are well-understood at graduation and translated into nursing practice [3,7,12].

Overall, students in this study demonstrated poor knowledge of pressure injury prevention with age and number of previous clinical training experiences significantly influencing scores. Many students could not correctly define pressure injury (caused by a lack of oxygen), consistent with previous studies that suggest students do not understand the aetiology of pressure injury [3,24,25]. Students could not correctly grade a pressure injury and students also struggled to identify patient needs with specific types of pressure injuries. The highest scores across all cohorts were related to pressure injury nutrition which also concurs with other studies [24,25]. Whilst cohort knowledge score differences were statistically significant, it was evident that each cohort had strengths and weaknesses. The 2016 cohort performed slightly more accurately in the nutrition knowledge as well as preventative measures; the 2017 cohort performed more accurately in pressure injury classification and observation; and the 2018 cohort obtained higher scores in the pressure injury risk assessment themes. This finding suggests that there was no consistent improvement over each year per theme, despite the introduction of additional educational interventions. However, the lower age of participants in the 2018 cohort combined with the lack of clinical experiences and clinical training units undertaken may have contributed to the lower scores in this cohort. A higher age was associated with better undergraduate academic performance in health studies [26], and access to and uptake of clinical experiences and clinical training units allow students more exposure to real-life scenarios, resulting in improved educational outcomes including knowledge retention [27,28].

Overall attitude scores were consistently high across the three cohorts with the 2018 cohort having a higher percentage of correct responses on average across the five themes, despite having a younger group and less male students, clinical experience, or exposure to training units, which is inconsistent with findings from other studies where more exposure to clinical training and higher age were linked to more favourable attitudes toward pressure injury prevention [12]. In this study, this finding may be attributed to the additional online and simulation educational materials provided to students and the way in which learning opportunities were delivered. A combination of active and reflective delivery methods has been shown to be more effective than only reflective learning strategies for nursing students [29,30]. This finding is also consistent with the 2016 cohort where only one form of education was delivered, and students scored lowest across all age groups, sexes, and number of clinical training units and clinical experience.

This research found that students reported high levels of responsibility for pressure injury prevention across all three cohorts, suggesting that there is an understanding of the important role of nurses in preventing pressure injuries. Self-reported confidence in the effectiveness of prevention was also high with most students, recognising that pressure injuries are preventable. However, similar to the findings of other studies, low knowledge of pressure injury prevention and a positive attitude may not suffice in clinical situations [7]. Developing capability in identification of pressure injuries, their causes, and the best preventative measures to reduce the amount of pressure on prominences of patients is necessary to ensure high-quality and safe care.

This research found that simulation and clinical practice improved knowledge for prevention and management of pressure injury. Mazzo and colleagues [31] indicated that high-quality clinical scenario simulation can positively support learning about pressure injury prevention and management. Additionally, studies [3,13] have specified that improved visual discrimination and opportunities to use clinical reasoning could improve educational outcomes. Kara and co-workers [12] suggested that more time in the classroom, in simulation, and in clinical practice needs to be dedicated to pressure injury prevention content. This research provides opportunity to invigorate nursing curricula to harness high attitude scores of student nurses and translate knowledge about pressure injury prevention into action [13,29] by consolidating theoretical knowledge during simulation and clinical practice experiences. Promoting behaviour change through active delivery methods [13,30] can address the knowledge translation gap that can promote learning about pressure injury [7].

### 4.1. Limitations

Limitations of this study include surveying separate cohorts of students with different demographics, which may have confounded the educational findings of this research. There are further potential confounding issues due the inconsistency of student completion of clinical units and previous awareness of pressure injury prevention due to family or employment contexts prior to provision of additional educational interventions. This research was undertaken prior to the COVID-19 pandemic whereby educational preparation pivoted to include more online learning, so students may now be more accepting of online learning packages to augment learning in simulation or clinical experiences than prior to the pandemic.

### 4.2. Recommendations

The findings of this study may be used to invigorate undergraduate nursing curriculum development by including a range of targeted online and simulation [31] learning interventions for students to tailor specific educational programs [3] and therefore improve the management and prevention of pressure injuries in clinical settings to promote work-readiness at graduation.

## 5. Conclusions

This research found that nursing student knowledge about pressure injury remained low regardless of the type of educational interventions available to support learning. However, attitudes towards pressure injury prevention improved when additional online and simulation resources were provided. Completion of one clinical unit, being more than 25 years of age, and male sex also improved knowledge about pressure injury prevention. These findings support other studies suggesting that all students, especially younger students, need educational preparation prior to undertaking clinical training units, which can be consolidated during clinical experiences. Further, targeted educational preparation about how to assess risk, undertake observation, and understand aetiology, as well as to develop strategies to prevent pressure injury or shear needs to be included in all undergraduate nursing curricula. This three-year intervention study showed that additional targeted educational interventions can make a difference to nurses’ attitudes about pressure injury; however, what interventions are most effective to translate knowledge into action requires further investigation.

## Figures and Tables

**Table 1:** Demographics (*n* = 1102).

Characteristic	Category	2016 *n* = 436	2017 *n* = 320	2018 *n* = 346	
		*n* (%)	*n* (%)	*n* (%)	*p*
*Age, mean (SD)*		25.8 (8.7)	26.1 (7.5)	24.8 (8.0)	0.074
**Age Group**					
	<20	108 (25.2)	81 (25.6)	116 (33.9)	<0.001
	20–25	168 (39.3)	90 (28.5)	118 (34.5)	
	>25	152 (35.5)	145 (45.9)	108 (31.6)	
**Sex**					
	Male	57 (13.3)	61 (19.2)	53 (15.3)	0.086
	Female	373 (86.7)	257 (80.8)	293 (84.7)	
**Number Clinical Training Units**					
	0	220 (50.5)	300 (93.8)	324 (93.6)	<0.001
	1	116 (26.6)	13 (4.1)	12 (3.5)	
	>1	100 (22.9)	7 (2.2)	10 (2.9)	
**Clinical Experiences**					
	0	220 (50.5)	300 (93.8)	324 (93.6)	<0.001
	1+	216 (49.5)	20 (6.3)	22 (6.4)	

**Table 2:** Pressure Injury Prevention Knowledge mean scores by demographic category (for participants that answered >= 24 questions).

		2016 *n* = 403	2017 *n* = 292	2018 *n* = 312	
Characteristic *n* = 1037	Category	Mean (SD)	Mean (SD)	Mean (SD)	*p*
Overall		12.86 (2.77)	13.18 (2.95)	12.59 (2.98)	0.040 *
Age	<20	12.29 (2.44)	13.30 (2.33)	12.92 (2.78)	0.013 *
	20–25	12.61 (2.82)	13.28 (3.28)	12.11 (2.76)	
	>25	13.66 (2.76)	13.15 (3.02)	12.75 (3.39)	
Sex	F	12.53 (2.78)	13.25 (2.86)	12.59 (2.98)	0.700
	M	12.91 (2.78)	13.22 (2.93)	12.58 (3.14)	
Number clinical training units	0	12.17 (2.77)	13.22 (2.97)	12.69 (2.94)	0.023 *
	1	13.38 (2.32)	12.38 (3.12)	12.10 (3.14)	
	>1	13.69 (2.86)	13.60 (0.55)	10.10 (3.28)	

* Denotes statistically significant difference.

**Table 3:** Knowledge responses by Theme 1.

Item	2016 *n* = 436	2017 *n* = 320	2018 *n* = 346	
	*n* (%)	*n* (%)	*n* (%)	*p*
***Theme 1:*** Pressure Injury Aetiology and Development				
**Which statement is correct:**				
Malnutrition causes pressure injuries.	150 (37.6)	115 (39.9)	87 (27.4)	0.005 **
A lack of oxygen causes pressure injuries. *	76 (19.0)	56 (19.4)	60 (18.9)	
Moisture causes pressure injuries:	173 (43.4)	117 (40.6)	171 (53.8)	
**Extremely thin patients are more at risk of developing a pressure injury than obese patients.**	177 (41.7)	165 (53.4)	190 (56.5)	
The contact area involved is small and thus the amount of pressure higher *	166 (39.4)	126 (40.0)	142 (42.3)	<0.001 **
The pressure is less extensive because the body weight of those patients < obese patients	93 (22.1)	107 (34.0)	100 (29.8)	
The risk of developing a vascular disorder is higher for obese patients and increases risk of PI	162 (38.5)	82 (26.0)	94 (28.0)	
**What happens when a patient, sitting in bed in a semi-upright position (60 degrees), slides down?**				
Pressure increases when the skin sticks to the surface.	87 (20.7)	53 (17.0)	47 (14.0)	0.046 **
Friction increases when the skin sticks to the surface.	186 (44.3)	124 (39.7)	155 (46.3)	
Shearing increases when the skin sticks to the surface. *	147 (35.0)	135 (43.3)	133 (39.7)	
**Which statement is correct:**				
Soap can dehydrate skin and thus the risk of PI	38 (9.2)	31 (10.0)	40 (12.2)	0.112
Moisture from urine, faeces, or wound drainage causes PI	189 (45.5)	114 (36.8)	135 (41.2)	
Shear is the force which occurs when the body slides and the skin sticks to the surface *	188 (45.3)	165 (53.2)	153 (46.6)	
**Which statement is correct:**				
Recent weight loss which has brought a patient below their ideal weight increases PI risk *	282 (65.7)	228 (73.5)	246 (73.0)	0.082
Very obese patients using medication that decreases the peripheral circulation not at risk of PI	100 (23.3)	50 (16.1)	58 (17.2)	
Poor nutrition and age have no impact on tissue tolerance when normal weight	47 (11.0)	32 (10.3)	33 (9.8)	
**There is NO relationship between pressure injury risk and:**				
Age	95 (22.2)	66 (21.4)	94 (28.3)	0.226
Dehydration	37 (8.6)	24 (7.8)	27 (8.1)	
Hypertension *	296 (69.2)	219 (70.9)	211 (63.6)	

* These are the correct answers. ** Denotes statistically significant difference. Bold statements are the questions asked in the survey.

**Table 4:** Knowledge responses by Theme 2.

Item	2016 *n* = 436	2017 *n* = 320	2018 *n* = 346	
	*n* (%)	*n* (%)	*n* (%)	*p*
***Theme 2:*** * Pressure Injury Classification and observation*				
**Which statement is correct:**				
A pressure injury extending down to the fascia is a grade 3 PI *	101 (23.7)	81 (26.2)	102 (30.4)	0.246
A pressure injury extending through the underlying fascia is a grade 3 PIs	217 (50.8)	145 (46.9)	158 (47.2)	
A grade 3 pressure injury is always preceded by a grade 2 PI	109 (25.5)	83 (26.9)	75 (22.4)	
**Which statement is correct:**				
A blister on a patient’s heel is always a grade 2 PI	131 (30.8)	79 (25.3)	115 (33.8)	0.010 **
All grades (1, 2, 3 and 4) of PIs involve loss of skin layers	87 (20.5)	47 (15.1)	49 (14.4)	
When necrosis occurs, it is a grade 3 or grade 4 PI *	207 (48.7)	186 (59.6)	176 (51.8)	
**Which statement is correct:**				
Friction or shear may occur when moving a patient in bed. *	343 (81.9)	264 (84.3)	276 (80.9)	0.833
A superficial lesion, preceded by non-blanchable erythema is probably a friction lesion	53 (12.6)	34 (10.9)	44 (12.9)	
A kissing ulcer (copy lesion) is caused by pressure and shear	23 (5.5)	15 (4.8)	21 (6.2)	
**In a sitting position, pressure injuries are most likely to develop on:**				
Pelvic area, elbow and heel. *	261 (60.6)	200 (62.7)	178 (53.0)	0.050
Knee, ankle and hip.	29 (6.7)	23 (7.2)	37 (11.0)	
Hip, shoulder and heel.	141 (32.7)	96 (30.1)	121 (36.0)	
**Which statement is correct:**				
All patients at risk of pressure injuries should have a systematic once a week	111 (26.2)	87 (28.3)	101 (30.8)	0.570
The skin of patients seated in a chair, who cannot move themselves should be inspected every 2–3 h	224 (53.0)	149 (48.5)	160 (48.8)	
The heels of patients who lie on a pressure redistributing surface should be observed at least once a day *	88 (20.8)	71 (23.1)	67 (20.4)	

* These are the correct answers. ** Denotes statistically significant difference. Bold statements are the questions asked in the survey.

**Table 5:** Knowledge responses by Theme 3.

Item	2016 *n* = 436	2017 *n* = 320	2018 *n* = 346	
	*n* (%)	*n* (%)	*n* (%)	*p*
***Theme 3:*** Pressure Injury Risk Assessment				
**Which statement is correct:**				
Risk assessment tools identify all high risk patients in need of prevention	109 (25.3)	82 (26.4)	57 (17.1)	0.030 **
The use of risk assessment scales reduces the cost of prevention	31 (7.2)	26 (8.4)	33 (9.9)	
A risk assessment scale may not accurately predict the risk of developing new PIs *	290 (67.4)	203 (65.3)	243 (73.0)	
**Which statement is correct:**				
The risk of pressure injury development should be assessed daily in all nursing homes	152 (35.8)	80 (26.0)	91 (27.4)	0.013 **
Absorbing pads should be placed under the patient to minimise risk of PI development	43 (10.1)	36 (11.7)	49 (14.8)	
A patient with a history of pressure injuries runs a higher risk of developing new PIs *	229 (54.0)	192 (62.3)	192 (57.8)	

* These are the correct answers. ** Denotes statistically significant difference. Bold statements are the questions asked in the survey.

**Table 6:** Knowledge responses by Theme 4.

Item	2016 *n* = 436	2017 *n* = 320	2018 *n* = 346	
	*n* (%)	*n* (%)	*n* (%)	*p*
***Theme 4:*** * Pressure Injuries and nutrition*				
**Which statement is correct:**				
Malnutrition causes pressure injuries.	42 (10.0)	31 (9.7)	27 (8.0)	0.001 **
The use of nutritional supplements can replace expensive preventative measures	4 (1.0)	10 (3.1)	22 (6.5)	
Optimising nutrition can improve the patient’s general physical condition which may reduce risk of PIs *	375 (89.1)	278 (87.1)	288 (85.5)	

* These are the correct answers. ** Denotes statistically significant difference. Bold statements are the questions asked in the survey.

**Table 7:** Knowledge responses by Theme 5.

Item	2016 *n* = 436	2017 *n* = 320	2018 *n* = 346	
	*n* (%)	*n* (%)	*n* (%)	*p*
***Theme 5:*** * Preventative measures to reduce the amount of pressure/shear*				
**The sitting position with the lowest amount of pressure between the body and the seat is:**				
An upright sitting position, with both feet resting on a footrest	145 (33.9)	89 (28.3)	91 (26.6)	0.043 **
An upright sitting position, with both feet resting on the floor	148 (34.6)	111 (35.4)	147 (43.0)	
A backwards sitting position, with both legs resting on a footrest *	135 (31.5)	114 (36.3)	104 (30.4)	
**Which repositioning scheme reduces pressure injury risk the most?**				
Supine position—side 90 degrees lateral position—supine	147 (34.7)	90 (29.7)	96 (28.7)	0.459
Supine position—side 30 degrees lateral position—side 30 *	128 (30.2)	99 (32.7)	109 (32.6)	
Supine position—side 30 degrees lateral position—sitting	149 (35.1)	114 (37.6)	129 (38.6)	
**Which statement is correct:**				
Patients who are able to change position while sitting should be taught to shift their weight minimum every 60 min while sitting *	333 (78.5)	234 (74.1)	219 (65.2)	<0.001 **
In a side lying position, the patient should be at a 90 degrees angle with the bed	23 (5.4)	29 (9.2)	49 (14.6)	
Shearing forces affect a patients sacrum maximally when the head of the bed is positioned at 30 degrees	68 (16.0)	53 (16.8)	68 (20.2)	
**If a patient is SLIDING down in a chair, the magnitude of pressure at the seat can be reduced the most by:**				
A thick air cushion *	113 (26.5)	91 (29.3)	95 (27.7)	0.388
A donut shaped foam cushion.	181 (42.4)	128 (41.2)	126 (36.7)	
A gel cushion.	133 (31.1)	92 (29.6)	122 (35.6)	
**For a patient at risk of developing a pressure injury, a visco-elastic foam mattress:**				
Reduces the pressure sufficiently and does not need to be combined with repositioning	71 (16.7)	50 (16.1)	48 (14.3)	0.331
Has to be combined with repositioning every 2 h.	198 (46.5)	125 (40.2)	153 (45.5)	
Has to be combined with repositioning every 4 h *	157 (36.9)	136 (43.7)	135 (40.2)	
**A disadvantage of a water mattress is:**				
Shear at the buttocks increases.	135 (31.5)	95 (30.4)	100 (29.8)	0.606
Pressure at the heels increases.	63 (14.7)	59 (18.8)	61 (18.2)	
Spontaneous small body movements are reduced. *	230 (53.7)	159 (50.8)	175 (52.1)	
**When a patient is lying on a pressure reducing foam mattress** **…**				
Elevation of the heels is not necessary.	39 (9.2)	24 (7.7)	35 (10.3)	0.233
Elevation of the heels is important. *	169 (39.8)	109 (34.8)	112 (33.0)	
He or she should be checked for ‘bottoming out’ at least twice a day	217 (51.1)	180 (57.5)	192 (56.6)	

* These are the correct answers. ** Denotes statistically significant difference. Bold statements are the questions asked in the survey.

**Table 8:** Knowledge responses by Theme 6.

Item	2016 *n* = 436	2017 *n* = 320	2018 *n* = 346	
	*n* (%)	*n* (%)	*n* (%)	*p*
Theme 6: *Preventative measures to reduce the duration of pressure/shear*				
**Repositioning is an active preventive measure because…**				
The magnitude of pressure and shear will be reduced.	66 (15.5)	43 (13.7)	42 (12.5)	0.661
The amount and the duration of pressure and shear will be reduced	217 (51.1)	165 (52.4)	169 (50.1)	
The duration of pressure and shear will be reduced. *	142 (33.4)	107 (34.0)	126 (37.4)	
**Fewer patients will develop a pressure injury if…**				
Food supplements are provided.	35 (8.5)	37 (12.1)	44 (13.3)	0.173
The areas at risk are massaged.	64 (15.5)	51 (16.7)	43 (13.0)	
Patients are mobilised. *	313 (76.0)	217 (71.1)	244 (73.7)	
**Which statement is correct:**				
Patients at risk lying on a non pressure reducing foam mattress should be repositioned every 2 h *	245 (58.8)	177 (56.9)	146 (43.8)	<0.001 **
Patients at risk lying on an alternating air mattress should be repositioned every 4 h	106 (25.4)	73 (23.5)	97 (29.1)	
Patients at risk lying on a visco-elastic foam mattress should be repositioned every 2 h	66 (15.8)	61 (19.6)	90 (27.0)	
**When a patient is lying on an alternating pressure air mattress, the prevention of heel pressure injuries includes:**				
No specific preventive measures.	40 (9.6)	33 (10.6)	44 (13.2)	0.342
A pressure reducing cushion under the heels.	143 (34.2)	120 (38.5)	120 (35.9)	
A cushion under the lower legs elevating the heels. *	235 (56.2)	159 (51.0)	170 (50.9)	
**If a bedridden patient cannot be repositioned, the most appropriate pressure injury prevention is…**				
A pressure redistributing foam mattress.	164 (38.8)	128 (41.7)	154 (45.8)	0.084
An alternating pressure air mattress. *	233 (55.1)	151 (49.2)	153 (45.5)	
Local treatment of the risk areas with zinc paste.	26 (6.1)	28 (9.1)	29(8.6)	

* These are the correct answers. ** Denotes a statistically significant difference. Bold statements are the questions asked in the survey.

**Table 9:** Attitude mean scores by demographic category (for participants that answered >= 12 questions).

		2016 *n* = 366	2017 *n* = 283	2018 *n* = 310	
Characteristic *n* = 989	Category	Mean (SD)	Mean (SD)	Mean (SD)	*p*
Overall		30.29 (2.78)	31.11 (3.06)	31.11 (2.98)	<0.001 *
Age	<20	29.85 (2.51)	30.76 (2.86)	30.60 (2.42)	0.001 *
	20–25	30.09 (2.91)	30.70 (2.81)	31.04 (2.50)	
	>25	30.74 (2.75)	31.56 (3.29)	31.67 (3.80)	
Sex	F	31.08 (2.61)	30.89 (2.98)	31.11 (2.98)	<0.001 *
	M	30.17 (2.81)	31.11 (3.07)	31.66 (3.56)	
Number clinical training	units	30.35 (2.79)	30.95 (2.80)	31.05 (2.71)	0.014 *
	0				
	1	30.42 (2.69)	34.00 (6.21)	33.10 (3.70)	
	>1	30.01 (2.87)	32.17 (2.40)	30.63 (7.98)	
Number clinical training units	0	30.35 (2.79)	33.39 (5.24)	31.11 (2.98)	0.012 *
	1+	30.23 (2.77)	31.11 (3.06)	30.84 (2.78)	

* Denotes statistically significant difference.

**Table 10:** Attitude responses.

Item	2016 *n* = 436	2017 *n* = 320	2018 *n* = 346	
	*n* (%)	*n* (%)	*n* (%)	*p*
***Theme 1: Personal competency to prevent PIs***				
**I feel confident in my ability to prevent PIs**				
Disagree	188 (44.2)	108 (34.6)	123 (36.4)	0.015 *
Agree	237 (55.8)	204 (65.4)	215 (63.6)	
**I am well trained to prevent PIs**				
Disagree	247 (58.3)	144 (46.6)	146 (43.5)	<0.001 *
Agree	177 (41.7)	165 (53.4)	190 (56.5)	
**PI prevention is too difficult. Others are better than I am**				
Disagree	266 (63.2)	186 (60.6)	192 (57.7)	0.304
Agree	155 (36.8)	121 (39.4)	141 (42.3)	
***Theme 2: Priority of PI prevention***				
**Too much attention goes to the prevention of PIs**				
Disagree	371 (88.1)	252 (82.4)	283 (84.7)	0.086
Agree	50 (11.9)	54 (17.6)	51 (15.3)	
**PI prevention is not that important**				
Disagree	407 (96.4)	290 (94.5)	306 (92.2)	0.037 *
Agree	15 (3.6)	17 (5.5)	26 (7.8)	
**PI prevention should be a priority**				
Disagree	26 (6.1)	19 (6.1)	20 (5.9)	0.994
Agree	402 (93.9)	295 (93.9)	319 (94.1)	
***Theme 3: Impact of pressure injuries***				
**A PI almost never causes discomfort for a patient**				
Disagree	407 (96.9)	281 (91.8)	310 (93.7)	0.010 *
Agree	13 (3.1)	25 (8.2)	21 (6.3)	
**The financial impact of PIs on a patient should not be exaggerated**				
Disagree	200 (48.3)	153 (50.5)	145 (43.9)	0.238
Agree	214 (51.7)	150 (49.5)	185 (56.1)	
**The financial impact of PIs on society is high**				
Disagree	102 (24.4)	47 (15.2)	57 (17.4)	0.004 *
Agree	316 (75.6)	263 (84.8)	271 (82.6)	
***Theme 4: Responsibility in pressure injury prevention***				
**I am not responsible if a PI develops in my patients**				
Disagree	384 (91.6)	274 (90.1)	300 (90.6)	0.768
Agree	35 (8.4)	30 (9.9)	31 (9.4)	
**I have an important task in PI prevention**				
Disagree	20 (4.7)	9 (2.9)	12 (3.6)	0.431
Agree	405 (95.3)	302 (97.1)	323 (96.4)	
***Theme 5: Confidence in the effectiveness of prevention***				
**PIs are preventable in high risk patients**				
Disagree	34 (8.3)	31 (10.2)	25 (7.5)	0.452
Agree	374 (91.7)	272 (89.8)	309 (92.5)	
**PIs are almost never preventable**				
Disagree	370 (92.5)	269 (88.8)	298 (92.0)	0.191
Agree	30 (7.5)	34 (11.2)	26 (8.0)	

Bold statements are the questions asked in the survey. * Denotes statistically significant difference. (Disagree = Strongly disagree + Disagree; Agree = Strongly agree + agree).

## Data Availability

The data presented in this study are available on request from the corresponding author. The data are not publicly available due to the ethical approval restrictions.
